# Reliability and validity study of the Spanish adaptation of the “Student Satisfaction and Self-Confidence in Learning Scale” (SCLS)

**DOI:** 10.1371/journal.pone.0255188

**Published:** 2021-07-23

**Authors:** Mariona Farrés-Tarafa, David Bande, Juan Roldán-Merino, Barbara Hurtado-Pardos, Ainoa Biurrun-Garrido, Lorena Molina-Raya, Marta Raurell-Torredà, Irma Casas, Urbano Lorenzo-Seva

**Affiliations:** 1 Campus Docent, Sant Joan de Déu-Fundació Privada, School of Nursing, University of Barcelona, Barcelona, Spain; 2 Research Group GIES (Grupo de investigación en Enfermería, Educación y Sociedad), Barcelona, Spain; 3 Member Research Group GRISIMula (Grupo emergente 2017 SGR 531; Grupo en Recerca Enfermera en Simulación), Barcelona, Spain; 4 Secretaria Research Group GRISCA (Grupo en Recerca Enfermera en Simulación en Cataluña y Andorra), Barcelona, Spain; 5 Servicio Anestesiología, Reanimación y Tratamiento del dolor, Parc de Salut Mar, Barcelona, Spain; 6 Research Group GEIMAC (Consolidated Group 2017-1681: Group of Studies of Invarianza of the Instruments of Measurement and Analysis of Change in the Social and Health Areas), Barcelona, Spain; 7 Coordinator Research Group GIRISAME (International Researchers Group of Mental Health Nursing Care), Barcelona, Spain; 8 Member Research Group GRIN (Grupo consolidado de recerca Infermeria, SRG:664), Barcelona, Spain; 9 Universidad de Barcelona, Barcelona, Spain; 10 Presidenta Sociedad Española de Enfermería Intensiva y Unidades Coronarias (SEEIUC), Madrid, Spain; 11 President Research Group GRISIMula (Grupo emergente 2017 SGR 531; Grupo en Recerca Enfermera en Simulación), Barcelona, Spain; 12 Universitat Autònoma de Barcelona, Barcelona, Spain; 13 Preventive Medicine Service, Hospital Germans Trias i Pujol, Badalona, Spain; 14 Research Group Innovation in Respiratory Infections and Tuberculosis Diagnosis (Group Consolidat 2017 SGR 494); 15 Universitat Rovira I Virgili, Tarragona, Spain; 16 ResearcherID: Lorenzo-Seva, U.G-4228-2011; Murcia University, SPAIN

## Abstract

The European Higher Education Area (EHEA) recommends the use of new educational methodologies and the evaluation of student satisfaction. Different instruments have been developed in Spain to evaluate different aspects such as clinical decisions and teamwork, however no instruments have been found that specifically evaluate student self-confidence and satisfaction during clinical simulation. The aim was to translate the Student Satisfaction and Self-Confidence in Learning Scale (SCLS) questionnaire into Spanish and analyse its reliability and validity and understand the level of satisfaction and self-confidence of nursing students with respect to learning in clinical simulations. The study was carried out in two phases: (1) adaptation of the questionnaire into Spanish. (2) Cross-sectional study in a sample of 489 nursing students. The reliability and exploratory and confirmatory factorial analyses were performed. To analyse the relationship of the scale scores with the socio-demographic variables, the Fisher Student T-test or the ANOVA was used. The scale demonstrated high internal consistency reliability for the total scale and each of its dimensions. Cronbach’s alpha was 0.88 (0.83 to 0.81) for each of the dimensions. The exploratory and confirmatory factor analysis showed that both the one-dimensional and two-dimensional models were acceptable. The results showed average scores above 4 for both dimensions. The SCLS-Spanish translation demonstrated evidence of its validity and reliability for use to understand the level of satisfaction and self-confidence of nursing students in clinical simulation. Clinical simulations help students to increase their levels of confidence and satisfaction, enabling them to face real scenarios in clinical practice.

## Introduction

The complexity of real world medical practice, high levels of patient acuity and requirements to mitigate risk and maximise safety and quality of care delivery mean that hospitals cannot maintain old on-the-job trial and error learning methods. Experimental learning and the practice of core skills is therefore now undertaken the safer setting of the simulation environment [1]. In the last decade, the use of simulation as a teaching-learning method has increased worldwide because it allowed the repetition (deliberate practice), it allowed to practice clinical cases that may not be applicable to clinical practice (variability of clinical practice) or that if they are seen they give the student a passive role (student-centred simulation) [2].

Systematic reviews have shown that Human Patient Simulation (HPS), compared to traditional educational methods, has superior results and improves student performance [3, 4].

Simulated-based learning (SBL) can improve learning compared to traditional methodologies, and its is especially effective in undergraduate students [5].

It aims to train their technical skills (procedures) and non-technical skills (decision-making, leadership, critical thinking, communication and teamwork, situational awareness, safe practice, adverse event minimization/mitigation and professionalism) [6] that will help them to transfer the knowledge learned to clinical practice. Non-technical skills can help newly qualified nurses to better understand their own role as a nurse, to know what others expect from them in professional practice, to improve their self-confidence and gain self-awarenness of their weaknesses [7]. Various studies [8–12] affirm that nursing students consider simulation to be an important methodology for their learning, that increases their satisfaction and that performing simulation activities consecutively increases their self-confidence, a sense of security which is soundly-based on the nurses ’awareness of their own capability’ which can contribute to reducing the theory-practice gap [13] On the other hand, by increasing self-confidence, it also decreases the anxiety that clinical practice can cause [14].

Therefore, it is necessary to know the level of satisfaction and degree of self-confidence that this teaching methodology generates in the student during their university education.

Due to the need to understand the degree of satisfaction and self-confidence generated by clinical simulation, in 2003, The National League for Nursing (NLN) together with Laerdal, created the instrument Nursing Student Satisfaction and Self-Confidence in Learning Scale (SCLS). It is a questionnaire consisting of 13 items [15], grouped into 2 dimensions: Satisfaction with Current Learning and self-confidence in learning during simulation.

Jeffries and Rizzolo (2007) [16] analysed the validity of the content and the reliability of the scale. The validity of the content was determined by nine clinical experts in nursing. In relation to the reliability of the scale, a Cronbach alpha of.94 was obtained for “Satisfaction with Current Learning” and an alpha of.87 for “Confidence in Learning” [16].

The (SCLS) is being used in different countries to assess the level of satisfaction and self-confidence of nursing students [17] and in multiple studies they conclude that simulation increases satisfaction and self-confidence in nursing students [11, 12, 18–20].

The improvement of student satisfaction with simulation activities motivates institutions to invest in this teaching strategy [18].

The reliability reported in the different studies performed was highly suitable with values greater than.85 [21–24].

Self-confidence is the belief that you have in your ability to succeed and that affects the effort that will be made when facing a task and the time you will persist for when a problem is encountered [25]. Therefore, it relates to the personal responsibility one has to achieve one’s goals. It is known that self-confidence is directly related to competence and success, and can interfere with the functionality of patient care. Self-confident nurses have developed their critical thinking, reflection, problem solving and decision-making skills better. Therefore, improving the self-confidence of nursing students can help in their training and thus reflect an improvement in the teaching and learning process [26].

Several instruments adequate for use to evaluate simulations have been identified in recent years [23, 27–30]. Instruments to evaluate different aspects of competencies, clinical decisions and teamwork have been developed in Spain [31–33], but only one instrument has been located that evaluates satisfaction [34]. It consists of 38 items and 8 dimensions (simulation utility, characteristics of cases and applications, communication, self-reflection on performance, increased self-confidence, relation between theory and practice, facilities and equipment and negative aspects of the simulation). However, no instrument has been found that specifically evaluates self-confidence and satisfaction of students during the clinical simulation and that can be completed in a short time.

However, it is necessary to have rigorously validated rubrics in Spain that can evaluate the effects of simulation-based activities. These instruments must be reliable and valid [35]. Since the creation of an instrument involves a high cost and requires a lot of time, the adaptation of existing instruments to another language offers several advantages. On the one hand, it reduces the cost of research, allowing the psychometric characteristics of the original instrument to be preserved and, on the other hand, it allows the results obtained to be compared, which are equally valid and reliable, with other national and international studies that have used the same instrument [36].

The objective of this study was to translate the Student Satisfaction and Self-Confidence in Learning Scale (SCLS) questionnaire into Spanish and analyse its reliability and validity and understand the level of satisfaction and self-confidence of nursing degree students with respect to learning in clinical simulations.

## Methods

### Study design

The study was carried out in two phases that consisted of the translation, adaptation to Spanish and validation of the scale, through a team of nurses skilled in simulation, followed by a descriptive and correlational study.

### Study setting and sample

The study sample consisted of 489 nursing students enrolled during the 2018–19 academic year. Non-probabilistic convenience sampling was used. The inclusion criteria were the following: to have performed some clinical simulation during the course and to have given their consent to participate in the study. Only those students who were not present at the time of handing out the surveys were excluded.

The recommendations of Comrey and Lee were followed to calculate the sample size [37] for validation studies, which suggest a graduated scale to determine the sample size: 100 = poor, 200 = fair, 300 = good, 500 = very good and 1,000 = excellent. In this study it was agreed to include approximately 500 participants from the different courses.

### Variables and source of information

All items related to the Nursing Student Satisfaction and Self-Confidence in Learning Scale (SCLS) questionnaire were picked as variables. It is a questionnaire consisting of 13 items, divided into 2 different dimensions (*Attitudes towards satisfaction with instruction* and *Self-confidence in learning in simulation* [15]. Each item is evaluated using a Likert scale with five possible answers, where 1) strongly disagree, 2) disagree, 3) undecided, 4) agree, and 5) strongly agree. The sum of the scores of all the items for each dimension gives us the estimated level of satisfaction and self-confidence of the student in the learning of clinical simulation. Other socio-demographic variables were also collected, such as age, sex, academic year, teaching shift, if they were working, work shift and if they had previous work experience in the health field.

### Procedure

There were two phases. The first phase consisted of translating and adapting the English version into Spanish through an independent bilingual English—Spanish, Spanish—English committee. The Standards for Educational and Psychological Testing [38] were followed throughout.

Initially 2 bilingual nurses, whose mother-tongue was Spanish carried out the translation from English to Spanish independently, without prior knowledge of the instrument or the objectives of the study. Then a committee of experts synthesized the 2 translations and created the first version of the instrument in Spanish. Later 2 nurses, whose mother-tongue was English independently retranslated the Spanish version to English.

The 2 versions obtained were compared with the original questionnaire by the same committee of experts. All agreed that the items in the Spanish version matched the original English version. However, to obtain the highest possible degree of semantic, idiomatic and conceptual equivalence, the expert committee decided to change the “teacher” to "Instructor/facilitator” on the understanding that, depending on the area of simulation where the learning is taking place, the teacher acquires one role or the other [39].

The expert committee consisted of two clinical simulation teachers accredited by the Boston Children’s Hospital, Simulator Program from Boston, two expert teachers in psychometrics and two nurses with advanced clinical practice experience.

Table 1 shows the semantic equivalence of items from English to Spanish, and the minimum and maximum scores for each dimension.

**Table 1:** Shows the semantic equivalence of items from English to Spanish that were metrically validated and distribution of the items in each dimension and minimum and maximum scores of the original student satisfaction and self-confidence in learning questionnaire.

English	Spanish	Scores D1
**D1. Satisfaction with Current Learning**	**D1. Satisfacción con el aprendizaje actual**	Items 1 to 5 Minimum score = 5 and Maximum score = 25
1. The teaching methods used in this simulation were helpful and effective.	1. Los métodos didácticos utilizados en la simulación fueron útiles y eficaces.	
2. The simulation provided me with a variety of learning materials and activities to promote my learning the medical.	2. La simulación me proporcionó una serie de materiales y escenarios de aprendizaje para impulsar mi aprendizaje durante mi formación	
3. I enjoyed how my instructor taught the simulation.	3. Me gustó cómo el instructor/facilitador desarrolló la actividad de simulación.	
4. The teaching materials used in this simulation were motivating and helped me to learn.	4. Los materiales didácticos utilizados en esta simulación fueron motivadores y me ayudaron a aprender.	
5. The way my instructor(s) taught the simulation was suitable to the way I learn.	5. La manera de enseñar la simulación por parte del instructor/facilitador se ajustó a mi manera de aprender.	
**D2. Self-confidence in Learning**	**D2. Confianza en uno mismo en el aprendizaje**	Scores D12
6. I am confident that I am mastering the content of the simulation activity that my instructors presented to me.	6. Estoy seguro de que domino el contenido de la actividad de simulación que los instructores me presentaron.	Items 6 to 13 Minimum score = 8 and Maximum score = 40
7. I am confident that this simulation covered critical content necessary for the mastery of medical surgical curriculum.	7. Estoy convencido de que esta simulación incluía contenidos fundamentales y necesarios para conseguir los objetivos de mi formación	
8. I am confident that I am developing the skills and obtaining the required knowledge from this simulation to perform necessary tasks in a clinical setting.	8. Estoy seguro de que esta simulación me permite desarrollar las competencias y obtener los conocimientos necesarios para realizar tareas necesarias en el ámbito clínico.	
9. My instructors used helpful resources to teach the simulation.	9. El instructor/ facilitador utilizó recursos útiles para enseñar la simulación.	
10. It is my responsibility as the student to learn what I need to know from this simulation activity.	10. Es mi responsabilidad como estudiante aprender lo que debo saber de esta actividad de simulación.	
11. I know how to get help when I do not understand the concepts covered in the simulation.	11. Sé cómo puedo obtener ayuda cuando no comprendo los conceptos tratados en la simulación.	
12. I know how to use simulation activities to learn critical aspects of these skills.	12. Sé cómo puedo utilizar las actividades de simulación para aprender aspectos fundamentales de estas competencias.	
13. It is the instructor’s responsibility to tell me what I need to learn of the simulation activity content during class time.	13. Es responsabilidad del instructor/facilitador explicarme lo que debo aprender del contenido de la actividad de simulación durante el prebriefing.	

Total questionnaire: Items from 1 to 13; Minimum score = 13 and Maximum score = 65.

Next, the pre-test was carried out on a sample of 10 nursing students from different courses and shifts. All of them concluded that it was easy to understand and required little time for completion (between 5 and 10 minutes). Subsequently, the questionnaire was administered to the nursing students included in the sample to analyse the psychometric properties of the Spanish version and the level of satisfaction and self-confidence in learning clinical simulation. The questionnaire was administered at the end of every simulation session.

### Data analysis

The internal consistency of the questionnaire was analysed using Cronbach’s alpha coefficient, considering acceptable values as those between.70 and.90 [40, 41].

A confirmatory factorial analysis was performed to analyse the validity of the construct (CFA) using the generalised least squares method. The overall fit quality was assessed using the indices: normed Chi-square, defined as the ratio between the value of the Chi-square and the number of degrees of freedom (χ2/df). Comparative Fit Index (CFI), Goodness of Fit Index (GFI), Adjusted Goodness of Fit Index (AGFI), Bentler Bonnet Normed Fit Index (BBNFI), Bentler Bonnet Non-Normed Fit Index (BBNNFI) and Root Mean Standard Error of Approximation (RMSEA). In order to consider a good overall fit, the criteria adopted were that of obtaining the following fit values: X^2^/df values between 2 and 6 [42]; CFI, GFI; AGFI; BBNFI and BBNNFI values ≥ 0.95 and RMSEA ≤ 0.05 [43–45].

CFA models were estimated using structural equation modelling (EQS 6.2 for Windows, Multivariate Software, Inc., Encino, CA, USA).

As the CFA suggested that a unidimensional factor solution could also be a plausive option, we computed Explained Common Variance (ECV) and Unidimensional Congruence (UniCo) indices to assess the degree of dominance of the general factor or closeness to uni-dimensionality [46]. ECV index essentially measures the proportion of common variance of the item scores that can be accounted for by the first canonical factor (i.e. the factor that explains most common variance). UniCo index is the congruence between the actual loading matrix and the loading matrix that would be obtained if the unidimensional model is true: the closer to the value of 1, the more the actual loading matrix looks like the unidimensional loading matrix. As for reference values, it has been proposed that ECV values should be in the range 0.70 to 0.85 if it is to be concluded that a solution can be treated as essentially unidimensional [46]. A value of UniCo larger than 0.95 suggests that data can be treated as essentially unidimensional [47]. Additionally, Optimal Implementation of Parallel Analysis (PA) was computed [48].

In order to explore the loading values of the items in a unidimensional solution, an exploratory factor analysis (EFA) was computed. Item scores were treated as ordered-categorical variables and the EFA was fitted to the inter-item polychoric correlation matrix [49]. The chosen fitting function was robust unweighted least squares, with mean-and-variance corrected fit statistics [50]. A single factor was extracted.

A descriptive analysis was carried out using frequencies and percentages, measures of central tendency and dispersion. To analyse the relationship of the scale scores with the socio-demographic variables, the Student T-test or the ANOVA was used.

Data analyses were performed using SPSS for Windows 22 (SPSS Institute, Chicago, IL, USA).

### Ethical considerations

The study was approved by the Clinical Investigation Ethics Committee of the San Joan de Déu Foundation with the assigned code CEIC PIC-42-19. The participants were informed about the authorship and purpose of the investigation and were ensured that all the data obtained would remain confidential. They freely gave their verbal and written consent to participate in the study as volunteers.

The translation has been completed with the permission of the National League for Nursing (NLN), but NLN is not responsible for its accuracy. Any request related to the translated instrument in Spanish must be addressed to NLN. More information about research instruments and copyright is available in NLN website [http://www.nln.org/professional-development-programs/research/tools-and-instruments]. NLN holds the copyright to the original (English language) and the translated instrument in Spanish.

## Results

### Demographic characteristics

A total of 489 students participated in the study. The mean age was 23.2 (SD 5.1), 82.4% being women. 60.1% of the students were enrolled in the morning study schedule. 60.5% of the students declared that they are currently working and of these, 43.6% had permanent employment. 67.2% of the students declared having work experience in the health field (Table 2).

**Table 2:** Sociodemographic characteristics of the study population.

Variables	n	%
**Age**	23.2 (SD 5.1)	
**Sex**		
male	86	17.6
female	403	82.4
**Study timetable**		
morning	294	60.1
afternoon	195	39.9
**Academic year**		
second	207	42.3
third	144	29.4
fourth	138	28.2
**Currently employed**		
yes	296	60.5
no	193	39.5
**Type of contract**		
permanent	129	43.6
temporary	167	56.4
**Work shift**		
mornings	76	25.7
afternoons	115	38.9
nights	36	12.2
rotating	69	23.3
**Years of grouped work experience**		
Less than 2 years	58	19.6
2–4 years	75	25.3
4–6 years	70	23.6
More than 6 years	93	31.4
**Healthcare work experience**		
yes	199	67.2
no	97	32.8

### Reliability

Cronbach’s alpha internal consistency coefficient for the total questionnaire was.885, and a value of.838 was obtained for dimension D1: Satisfaction with Current Learning and a value of.812 for dimension D2: Self-confidence in Learning. Cronbach’s alpha was also calculated for each item in the questionnaire and it was not observed that the exclusion of an item would improve the internal consistency of the questionnaire in general (Table 3).

**Table 3:** Internal consistency coefficient (Cronbach’s alpha) for the student satisfaction and self-confidence in learning questionnaire.

Item contents summarized	Mean	SD	Cronbach’s alpha		
			Total subscale	Total subscale without item	Total scale without item
**Satisfaction with Current Learning (total)**	4.2	0.053	0.838		
1. The teaching methods used in this simulation were helpful and effective.	4.1	0.68		0.646	0.873
2. The simulation provided me with a variety of learning materials and activities to promote my learning the medical. surgical curriculum.	4.3	0.67		0.667	0.873
3. I enjoyed how my instructor taught the simulation.	4.2	0.68		0.609	0.875
4. The teaching materials used in this simulation were motivating and helped me to learn.	4.1	0.75		0.601	0.875
5. The way my instructor(s) taught the simulation was suitable to the way I learn.	4.1	0.72		0.613	0.875
**Self-confidence in Learning (total)**	4.1	0.04	0.812		
6. I am confident that I am mastering the content of the simulation activity that my instructors presented to me.	3.7	0.73		0.490	0.881
7. I am confident that this simulation covered critical content necessary for the mastery of medical surgical curriculum.	4.2	0.76		0.641	0.873
8. I am confident that I am developing the skills and obtaining the required knowledge from this simulation to perform necessary tasks in a clinical setting.	4.1	0.75		0.663	0.872
9. My instructors used helpful resources to teach the simulation.	4.2	0.67		0.638	0.874
10. It is my responsibility as the student to learn what I need to know from this simulation activity.	4.3	0.76		0.484	0.881
11. I know how to get help when I do not understand the concepts covered in the simulation.	4.1	0.80		0.458	0.883
12. I know how to use simulation activities to learn critical aspects of these skills.	4.0	0.75		0.611	0.875
13. It is the instructor’s responsibility to tell me what I need to learn of the simulation activity content during class time.	4.1	0.78		0.351	0.889
Total questionnaire			0.885		

### Construct validity

The factorial structure was analysed by means of a confirmatory factorial analysis in which a 2-dimensional model identical to the structure of the original version was proposed.

**Fig 1:**
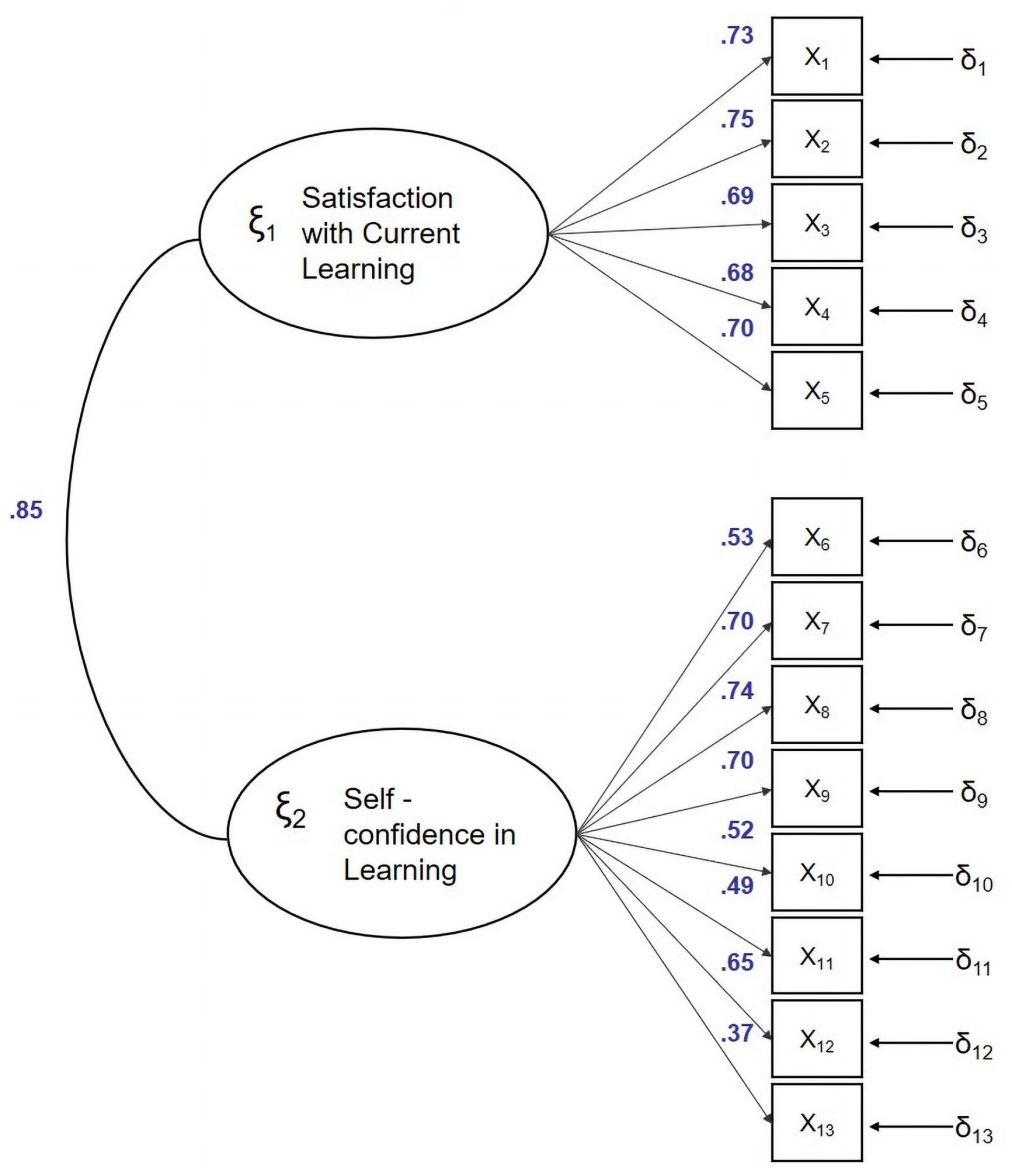
Standardized model parameters.

Parameter estimation was performed using the least squares method. Dimension 1 was the one with the greatest factorial load or saturation of its indicators. All saturations showed values larger than.30 (see Fig 1).

The Chi square test was statistically significant but the fit ratio was 4.28, so if it is between 2–6 the fit is reasonably good [51]. Likewise, both the rest of the absolute adjustment indices, as well as incremental adjustment and parsimony indexes analysed, presented the same trend, so it can be concluded that the model suitably fits (Table 4).

**Table 4:** Indices of goodness of fit of the confirmatory model.

INDEX	VALUE
BBNFI	0.954
BBNNFI	0.976
CFI	0.980
GFI	0.988
AGFI	0.982
RMSEA	0.039
α Cronbach	0.887
Goodness of fit test	χ^2^ = 274,234; gl = 64; *p* < 0.0001
Reason for fit	χ^2^ / gl = 4.28 between 2–6

BBNFI: Bentler Bonnet Normed Fit Index. BBNNFI: Bentler Bonnet Non-Normed Fit Index.

CFI: Comparative Fit Index. GFI: Goodness of Fit Index. AGFI: Adjusted Goodness of Fit Index. RMSEA: Root Mean Standard Error of Approximation.

As can be observed in Fig 1, CFA model was adjusted for a model were the two factors correlated among them.85. As this correlation is large, a unidimensional factor model could be expected to fit properly. The ECV value was.864, the UniCo value was.965: both values suggested that there is a strong dominant factor running through all the 13 items. In addition, the first eigenvalue accounted for 55.1% of the common variance (the second eigenvalue accounted for 10.7%), and PA suggested that the unidimensional solution is the most replicable.

Goodness of fit indices for the single factor model are printed in Table 5. As can be observed in the table, the fit is not so good as the two-dimensional model tested in CFA, but it is still acceptable. The loading values of the items in the EFA ranged between.41 (item 13) and.78 (item 2). Finally, Expected At Posteriori reliability [52] of the factor was.923.

**Table 5:** Indices of goodness of fit of the exploratory unidimension to the model.

INDEX	VALUE	95% confidence interval
CFI	0.978	0.968–0.989
GFI	0.981	0.974–0.989
AGFI	0.977	0.969–0.987
RMSEA	0.073	0.060–0.081
Goodness of fit test	χ^2^ = 55,383; *gl* = 42; *P* < 0.0001	
Reason for fit	χ^2^ / *gl* = 1.32	

CFI: Comparative Fit Index. GFI: Goodness of Fit Index. AGFI: Adjusted Goodness of Fit Index. RMSEA: Root Mean Standard Error of Approximation.

The conclusion is that both (unidimensional and bidimensional) models are acceptable. From a practical point of view, it means that researchers can compute the overall scale score (i.e., the score that is obtained using the responses to the all items), but also the score in two subscales (Satisfaction and Self-confidence) when a more detailed description of participant responses may be needed.

#### Personal attitudes about the instruction the student receives during their simulation activity (satisfaction with current learning and self-confidence in learning)

The average total score of the questionnaire was 54.4 (SD 6.2), with the minimum score being 19 and the maximum being 65. In order to compare the scores of the two dimensions, the average score of each dimension has been divided by the number of items that configure it. The scores were higher in dimension D1: Satisfaction with Current Learning than in D2: Self-confidence in Learning (Average 4.2, SD 0.5 and Average 4.1, SD 0.4 respectively) (Table 3).

When relating the total score of the questionnaire and the socio-demographic and labour variables of the students, only statistically significant differences were found with the teaching shift and the academic year. The total average score of the questionnaire was higher in the students who did the training in the morning shift and in the second-year students who had performed fewer clinical simulations (*p* = .019 and *p* = .032 respectively) (Table 6).

**Table 6:** Satisfaction and self-confidence of clinical simulation with respect to socio-demographic variables.

Variables	n	Score SCLS total		D1. Satisfaction with Current Learning		D2. Self-confidence in Learning	
		Mean	p	Mean	p	Mean	p
**Age**							
Under 21 years old	146	55.1	0.259	21.7	0.024	33.4	0.702^1^
21–23	197	54.0		20.9		33.1	
Over 23 years old	146	54.5		21.8		33.4	
**Sex**							
male	86	54.5	0.992	21.0	0.603	33.4	0.700^2^
female	403	54.5		21.2		33.2	
**Study timetable**							
morning	294	55.0	0.019	21.4	0.009	33.5	0.066^2^
afternoon	195	53.7		20.8		32.9	
**Academic year**							
second	207	55.3	0.032	21.7	0.0001	33.5	0.429 ^1^
third	144	53.6		20.5		33.0	
fourth	138	54.5		21.0		33.1	
**Currently employed**							
yes	296	54.8	0.208	21.3	0.373	33.5	0.175 ^2^
no	193	54.0		21.0		33.0	
**Type of contract**							
permanent	129	54.5	.512	21.2	0.747	33.3	0.428 ^2^
temporary	167	54.9		21.3		33.6	
**Work shift**							
mornings	76	54.8	.991	21.4	0.761	33.4	0.934^1^
afternoons	115	54.6		21.2		33.4	
nights	36	54.8		21.0		33.8	
rotating	69	54.8		21.5		33.3	
**Years of grouped work experience**							
Less than 2 years	58	53.8	0.324	20.8	0.371	32.9	0.435 ^1^
2–4 years	75	54.8		21.2		33.6	
4–6 years	70	55.6		21.6		33.9	
More than 6 years	93	54.6		21.3		33.3	
**Healthcare work experience**							
yes	199	54.7	0.948	21.2	0.382	33.5	0.597^2^
no	97	54.8		21.4		33.3	

Univariate analysis.

^1^ p value for ANOVA;

^2^ value for t student Fisher.

## Discussion

Firstly, the study aimed to evaluate the psychometric properties of the Nursing Student Satisfaction and Self-Confidence in Learning Scale (SCLS) questionnaire in nursing degree students from Spain. The results have shown that the psychometric properties are adequate in terms of internal consistency and the validity of the construct. An important aspect to highlight in this study is the sample size, in which 489 nursing students from different academic courses have participated, a sufficient sample to perform a confirmatory factorial analysis [37]. This figure is higher than that used in different studies in which the SCLS questionnaire has been validated [35, 53–56].

In relation to the reliability of the questionnaire, a Cronbach Alpha of.885 was obtained for the overall questionnaire and greater than.81 for the two dimensions that configure it: Satisfaction and self-confidence, considering these to be appropriate values [41].

This instrument has been translated into different languages and for different countries (Turkish, Chinese, Portuguese, and Norwegian), where Cronbach’s alpha was also greater than.75 in all cases for the total scale and for each dimension, except for the study conducted in Norway where it was 0.64 [55, 56].

In 2014 [17] conducted the first study, to learn the psychometric properties of the Nursing Student Satisfaction and Self-Confidence in Learning Scale (SCLS) questionnaire. In this study reliability was analysed and an exploratory factorial analysis was performed, where 2 dimensions were identified: satisfaction and self-confidence that reported reliability for each of them as.92 and.83, similar results to those found in our study.

With respect to the validity of the construct, Almeida et al. and Tosterud et al. [54, 55] performed an exploratory factorial analysis. In both studies they identified the two dimensions and they verified that the indices obtained fit the model properly [54–56].

In our study, a confirmatory factorial analysis was carried out using the generalised least squares method in order to determine if the scores reproduced the two-dimensional structure on which the original questionnaire is based. The confirmatory factorial analysis showed a bi-factorial model in which all the items presented an adequate factorial load. With respect to the fit indices analysed for the model, both the absolute fit indices: GFI (Goodness of Fit Index), RMSEA (Root Mean Square Error of Approximation), and the incremental fit indices: AGFI (Adjusted Goodness of Fit Index), BBNFI (Bentler Bonnet Normed Fit Index), BBNNFI (Bentler Bonnet Non Normed Fit Index), CFI (Comparative Fit Index) and the parsimony indices such as the normed Chi-square all present a good fit, so it can be concluded that the model suitably fits. These results are very similar to those found in the study conducted by Franklin and Chan [17, 53]. In addition, we computed an exploratory factor analysis and observed that the unidimensional factor solution is also acceptable for the test.

In the simulation, knowledge, performance in situ, the educational practices used, student satisfaction and self-assessment, as well as critical thinking ability and self-confidence can be assessed [8, 57]. A systematic review found that high-fidelity simulation improves knowledge and skills but not so much self-confidence [58]. In this study, the degree of satisfaction and self-confidence obtained by nursing students after the high-fidelity clinical simulation was evaluated. The results have shown average scores above 4 for both dimensions, which shows that students satisfactorily value simulation during academic training, these results are similar to those found in other studies that also use the SCLS to assess satisfaction and self-confidence [11, 12, 18, 19, 59]. In our sociocultural context, a study also showed that satisfaction with simulation is very high (96% totally agree/In agreement) and that simulation promotes self-confidence (80,6% totally agree/In agreement) [34]. In no study have statistically significant differences been found when relating socio-demographic variables to the total score of the questionnaire. However, in our study, the teaching shift and the academic year showed significant results. This may be because the profile of students in the afternoon shift is more demanding because the vast majority are working and the average age is higher.

### Limitations

Our study has certain limitations. First of all, we selected a sample of convenience from a single university in Barcelona, therefore, it is possible that our results cannot be generalised to all nursing students. However, the socio-demographic and work characteristics of the students in this study are similar to other universities in Spain.

Another limitation is that the degree of satisfaction was analysed at a given time. More research should be done to find out if self-confidence increases as the student performs more simulations.

## Conclusions

The SCLS-Spanish translation demonstrated evidence of its validity and reliability for use to understand the level of satisfaction and self-confidence of nursing students in clinical simulation. Likewise, simulation as an innovative teaching methodology will enable the student’s self-confidence and satisfaction to be assessed throughout their university education.

Clinical simulations help students to increase their levels of confidence and satisfaction, enabling them to face real scenarios in clinical practice better prepared and with more confidence.

## Supporting information

S1 File (XLSX)

## References

[1] Parker BA, Grech C. Authentic practice environments to support undergraduate nursing students’ readiness for hospital placements. A new model of practice in an on campus simulated hospital and health service. Nurse Educ Pract. 2018;33:47–54. doi: 10.1016/j.nepr.2018.08.012 30241029

[2] Bogossian FE, Cant RP, Ballard EL, Cooper SJ, Levett-Jones TL, McKenna LG, et al. Locating “gold standard” evidence for simulation as a substitute for clinical practice in prelicensure health professional education: A systematic review. J Clin Nurs. 2019;28(21–22):3759–75. doi: 10.1111/jocn.14965 31216367

[3] Jeffries PR, Rogers K. Evaluating simulations. In: Jeffries PR, editor. Simulation in Nursing Education: from Conceptualization to Evaluation National League for Nursing. New York; 2007. p. 87–103.

[4] Çalım Sİ, Ulaş SC, Demirci H, Tayhan EB. Effect of simulation training on students′ childbirth skills and satisfaction. Nurse Educ Pract. 2020 May;46:102808. doi: 10.1016/j.nepr.2020.102808 32521473

[5] Shin S, Park JH, Kim JH. Effectiveness of patient simulation in nursing education: Meta-analysis. Nurse Educ Today. 2015;35(1):176–82. doi: 10.1016/j.nedt.2014.09.009 25459172

[6] Lopreiato, J. O. (Ed.), Downing, D., Gammon, W., Lioce, L., Sittner, B., Slot, V., et al. Healthcare Simulation Dictionary. The Healthcare Simulation Dictionary. 2016.

[7] Raurell-Torredà M, Romero-Collado À, Bonmatí-Tomàs A, Olivet-Pujol J, Baltasar-Bagué A, Solà-Pola M, et al. Objective Structured Clinical Examination: An Assessment Method for Academic-Practice Partnerships. Clin Simul Nurs. 2018;8–16.

[8] Farrés-Tarafa M, Roldán-Merino J, Lorenzo-Seva U, Hurtado-Pardos B, Biurrun-Garrido A, Molina-Raya L, et al. Reliability and validity study of the Spanish adaptation of the “Educational Practices Questionnaire” (EPQ). PLoS One. 2020;15(9):e0239014. doi: 10.1371/journal.pone.0239014 32941464 PMC7497994

[9] El Naggar MA, Almaeen AH. Students’ perception towards medical-simulation training as a method for clinical teaching. J Pak Med Assoc. 2020;70(4):618–23. 32296205 10.5455/JPMA.6481

[10] Powers K. Bringing simulation to the classroom using an unfolding video patient scenario: A quasi-experimental study to examine student satisfaction, self-confidence, and perceptions of simulation design. Nurse Educ Today. 2020;86:104324. doi: 10.1016/j.nedt.2019.104324 31901748

[11] Lubbers J, Rossman C. Satisfaction and self-confidence with nursing clinical simulation: Novice learners, medium-fidelity, and community settings. Nurse Educ Today. 2017;48:140–4. doi: 10.1016/j.nedt.2016.10.010 27810632

[12] Zapko KA, Ferranto MLG, Blasiman R, Shelestak D. Evaluating best educational practices, student satisfaction, and self-confidence in simulation: A descriptive study. Nurse Educ Today. 2018;60:28–34. doi: 10.1016/j.nedt.2017.09.006 28987895

[13] Monaghan T. A critical analysis of the literature and theoretical perspectives on theory-practice gap amongst newly qualified nurses within the United Kingdom. Nurse Educ Today. 2015;35(8):e1–7. doi: 10.1016/j.nedt.2015.03.006 25862073

[14] Khalaila R. Simulation in nursing education: An evaluation of students’ outcomes at their first clinical practice combined with simulations. Nurse Educ Today. 2014;34(2):252–8. doi: 10.1016/j.nedt.2013.08.015 24060462

[15] Jeffries PR, Rizzolo MA. SUMMARY REPORT Project Title: Designing and Implementing Models for the Innovative Use of Simulation to Teach Nursing Care of Ill Adults and Children: A National, Multi-Site, Multi-Method Study Project Sponsors National League for Nursing and Laerdal Medi. 2006.

[16] Jeffries PR, Rizzolo M. Appendix A. Final report of the NLN/Laerdal simulation study. In: Jeffries PR, editor. Simulation in Nursing Education: from Conceptualization to Evaluation. New York; 2007. p. 145–59.

[17] Franklin AE, Burns P, Lee CS. Psychometric testing on the NLN Student Satisfaction and Self-Confidence in Learning, Simulation Design Scale, and Educational Practices Questionnaire using a sample of pre-licensure novice nurses. Nurse Educ Today [Internet]. Elsevier Ltd; 2014;34(10):1298–304. Available from: 10.1016/j.nedt.2014.06.011 25066650

[18] Bergamasco EC, Murakami BM, De Almeida Lopes Monteiro da Cruz D. Use of the Student Satisfaction and Self-Confidence in Learning (SSSCL) and the Simulation Design Scale (SDS) in nursing teaching: Experience report. Sci Med (Porto Alegre). 2018;28(3):1–5.

[19] Omer T. Nursing students’ perceptions of satisfaction and self-confidence with clinical simulation experience. J Educ Pract. 2016;7(5):131–8.

[20] Zhu F-F, Wu L-R. The effectiveness of a high-fidelity teaching simulation based on an NLN/Jeffries simulation in the nursing education theoretical framework and its influencing factors. Chinese Nurs Res. 2016;3(3):129–32.

[21] Butler KW, Veltre DE, Brady D. Implementation of Active Learning Pedagogy Comparing Low-Fidelity Simulation Versus High-Fidelity Simulation in Pediatric Nursing Education. Clin Simul Nurs. 2009;5(4):E129–36.

[22] Cantrell MA, Meakim C, Cash K. Development and Evaluation of Three Pediatric-based Clinical Simulation. Clin Simul Nurs. 2008;4(1):e21–8.

[23] Kardong-Edgren S, Adamson KA, Fitzgerald C. A Review of Currently Published Evaluation Instruments for Human Patient Simulation. Clin Simul Nurs. 2010;6(1):e25–35.

[24] Sittner BJ, Schmaderer M, Zimmerman L, Hertzog M, George B. Rapid Response Team Simulated Training for Enhancing Patient Safety (STEPS). Clin Simul Nurs. 2009;5(3):e119–27.

[25] Bandura A. Social foundations of thought and action: Social cognitive theory. Englewood Cliffs, New Jersey: Prentice Hall. 1986. 617 p.

[26] Pike T, O’Donnell V. The impact of clinical simulation on learner self-efficacy in pre-registration nursing education. Nurse Educ Today. 2010;30(5):405–10. doi: 10.1016/j.nedt.2009.09.013 19883960

[27] Oh PJ, Jeon KD, Koh MS. The effects of simulation-based learning using standardized patients in nursing students: A meta-analysis. Vol. 35, Nurse Education Today. 2015. p. e6–15. doi: 10.1016/j.nedt.2015.01.019 25680831

[28] Sigalet E, Donnon T, Grant V. Undergraduate students’ perceptions of and attitudes toward a simulation-based interprofessional curriculum: The kidSIM ATTITUDES questionnaire. Simul Healthc. 2012;7(6):353–8. doi: 10.1097/SIH.0b013e318264499e 22902608

[29] Levett-Jones T, Lapkin S. A systematic review of the effectiveness of simulation debriefing in health professional education. Vol. 34, Nurse Education Today. 2014. p. e58–e63. doi: 10.1016/j.nedt.2013.09.020 24169444

[30] Cant RP, Cooper SJ. Use of simulation-based learning in undergraduate nurse education: An umbrella systematic review. Nurse Educ Today. 2017;(49):63–71. 10.1016/j.nedt.2016.11.015 27902949

[31] Roldán-Merino J, Farrés-Tarafa M, Estrada-Masllorens JM, Hurtado-Pardos B, Miguel-Ruiz D, Nebot-Bergua C, et al. Reliability and validity study of the Spanish adaptation of the “Creighton Simulation Evaluation Instrument (C-SEI)”. Nurse Educ Pract. 2019;35:14–20. doi: 10.1016/j.nepr.2018.12.007 30640046

[32] Montejano-Lozoya R, Gea-Caballero V, Miguel-Montoya I, Juárez-Vela R, Sanjuán-Quiles Á, Ferrer-Ferrandiz E. Validation of a questionnaire designed to measure nursing student satisfaction with practical training. Rev Lat Am Enfermagem. 2019;27:e3206. doi: 10.1590/1518-8345.3102.3206 31826154 PMC6896794

[33] Raurell-Torredà M, Bonmatí-Tomás A, Lamoglia-Puig M, Zaragoza-García I, Farrés-Tarafa M, Roldán-Merino J, et al. Psychometric design and validation of a tool to assess the medication administration process through simulation in undergraduate nursing students. Nurse Educ Today. 2021;98. doi: 10.1016/j.nedt.2020.104726 33493925

[34] Alconero-Camarero AR, Romero AG, Sarabia-Cobo CM, Arce AM. “Clinical simulation as a learning tool in undergraduate nursing: Validation of a questionnaire.” Nurse Educ Today. 2016;39:128–34. doi: 10.1016/j.nedt.2016.01.027 27006044

[35] Unver V, Basak T, Watts P, Gaioso V, Moss J, Tastan S, et al. The reliability and validity of three questionnaires: The Student Satisfaction and Self-Confidence in Learning Scale, Simulation Design Scale, and Educational Practices Questionnaire. Contemp Nurse. 2017;53(1):60–74. doi: 10.1080/10376178.2017.1282319 28084900

[36] Escobar Bravo MÁ. Adaptación transcultural de instrumentos de medida relacionados con la salud. Enfermería Clínica. 2004;14(2):102–6.

[37] Comrey AL. A First Course in Factor Analysis. A First Course in Factor Analysis. 2013.

[38] Frey BB. Standards for Educational and Psychological Testing. In: The SAGE Encyclopedia of Educational Research, Measurement, and Evaluation. 2018.

[39] Roussin CJ, Weinstock P. SimZones. Acad Med. 2017;92(8):1114–20. doi: 10.1097/ACM.0000000000001746 28562455

[40] Cronbach LJ. Coeffiecient alpha and the internal structure of test. Psychometrika. 1951;16:297–334.

[41] Nunnally JC, Bernstein IH. The theory of measurement error. In: Psychometric Theory. 1994. p. 209–47.

[42] Hu L, Bentler P. Cutoff criteria for fit indexes in covariance structure analysis: Conventional criteria versus new alternatives. Struct Equ Model A Multidiscip J. 1999;6(1):1–55.

[43] Byrne BM. Structural Equation Modeling With AMOS. Structural Equation Modeling With AMOS. 2016.

[44] Brown TA. Confirmatory factor analysis for applied research. Second Edi. Confirmatory factor analysis for applied research. New York: The Guildford Press; 2015. 462 p.

[45] Schermelleh-Engel K, Moosbrugger H, Müller H. Evaluating the Fit of Structural Equation Models: Tests of Significance and Descriptive Goodness-of-Fit Measures. Methods Psychol Res Online. 2003;8(2):23–74.

[46] Ferrando, Lorenzo-Seva U. On the Added Value of Multiple Factor Score Estimates in Essentially Unidimensional Models. Educ Psychol Meas. 2019;79(2):249–71. doi: 10.1177/0013164418773851 30911192 PMC6425092

[47] Lorenzo-Seva U, ten Berge JMF. Tucker’s congruence coefficient as a meaningful index of factor similarity. Methodology. 2006;2(2):57–64.

[48] Timmerman ME, Lorenzo-Seva U. Dimensionality assessment of ordered polytomous items with parallel analysis. Psychol Methods. 2011;16(2):209–20. doi: 10.1037/a0023353 21500916

[49] Ferrando, Lorenzo-Seva U. Unrestricted item factor analysis and some relations with item response theory [Internet]. Department of Psychology, Universitat Rovira i Virgili, Tarragona; 2013. http://psico.fcep.urv.es/utilitats/factor

[50] Ferrando PJ, Lorenzo-Seva U. Program FACTOR at 10: Origins, development and future directions. Psicothema. 2017;29(2):236–40. doi: 10.7334/psicothema2016.304 28438248

[51] Rial A, Varela J, Abalo J, Lévy JP. El análisis factorial confirmatorio. In: Lévy JP, Varela J, editors. Modelización con estructuras de covarianzas en ciencias sociales: temas esenciales, avanzados y aportaciones especiales. España: Gesbiblo S. L.; 2006. p. 119–54.

[52] Ferrando PJ, Lorenzo-Seva U. A note on improving EAP trait estimation in oblique factor-analytic and item response theory models. Psicologica. 2016;37(2):235–47.

[53] Chan JCK, Fong DYT, Tang JJ, Pui Gay K, Hui J. The chinese student satisfaction and self-confidence scale is reliable and valid. Clin Simul Nurs. 2015;11(5):278–83.

[54] dos S Almeida RG, Mazzo A, Martins JCA, Baptista RCN, Girão FB, Mendes IAC. Validation to Portuguese of the scale of student satisfaction and self-confidence in learning. Rev Lat Am Enfermagem. 2015;23(6):1007–13. doi: 10.1590/0104-1169.0472.2643 26625990 PMC4663999

[55] Tosterud R, Petzäll K, Hedelin B, Hall-Lord ML. Psychometric testing of the norwegian version of the questionnaire, student satisfaction and self-confidence in learning, used in simulation. Nurse Educ Pract. 2014;14(6):704–8. doi: 10.1016/j.nepr.2014.10.004 25458231

[56] Jara VJ, Núñez CS. Clinical simulation in nursing: A scale to evaluate satisfaction and self-confidence in learning. Stud Health Technol Inform. 2018;250(2016):89–90. 29857393

[57] Roldán-Merino J, Farrés-Tarafa M, Estrada-Masllorens JM, Hurtado-Pardos B, Miguel-Ruiz D, Nebot-Bergua C, et al. Reliability and validity study of the Spanish adaptation of the “Creighton Simulation Evaluation Instrument (C-SEI)”. Nurse Educ Pract. 2019;35:14–20. doi: 10.1016/j.nepr.2018.12.007 30640046

[58] Haddeland K, Slettebø Å, Carstens P, Fossum M. Nursing Students Managing Deteriorating Patients: A Systematic Review and Meta-Analysis. Clin Simul Nurs. 2018;21:1–15.

[59] Haskell B, Thul S. Impact of a Standardized Patient Simulation on Behavioral Health Nurse Resident Confidence and Satisfaction in Learning. J Nurses Prof Dev. 2020;36(4):221–6. doi: 10.1097/NND.0000000000000620 32187085

